# Characteristics of Chinese women in need of enhanced sexual health attention and at risk of hypoactive sexual desire disorder

**DOI:** 10.1186/s12905-023-02357-5

**Published:** 2023-06-13

**Authors:** Lan Luo, Jingjing Huang, Huafang Li

**Affiliations:** 1 Shanghai Jiao Tong University School of Medicine, 600, Wan Ping Nan Lu, Shanghai, 200030 China; 2 Shanghai mental health center, Shanghai Jiao Tong university school of medicine, 600, Wan Ping Nan Lu, Shanghai, 200030 China; 3 Shanghai clinical research center for mental health, 600, Wan Ping Nan Lu, Shanghai, 200030 China; 4 Shanghai key laboratory of psychotic disorders, 600, Wan Ping Nan Lu, Shanghai, 200030 China

**Keywords:** Sexual health, Attitude to health, Sexual distress, Hypoactive sexual desire disorder, Women's health service

## Abstract

**Background:**

The target population for women’s sexual health services in China was unclear. To identify high-risk individuals with psychological barriers to sexual health-seeking behaviors and those at high risk of hypoactive sexual desire disorder (HSDD), we investigated correlates of Chinese women’s unwillingness to communicate sexual health, the shame of sexual health-related disorders, sexual distress, and HSDD.

**Methods:**

An online survey was conducted from April to July 2020.

**Results:**

We received 3443 valid responses online (effective rate 82.6%). Participants were mainly Chinese urban women of childbearing age (median 26 years old, Q1-Q3 23–30). Women who knew little about sexual health knowledge (aOR 0.42, 95%CI 0.28–0.63) and were ashamed (aOR 0.32–0.57) of sexual health-related disorders were less willing to communicate sexual health. Age (aOR 4.29, 95%CI 2.26–8.17), low income (aOR 1.52–2.11), family burden (aOR 1.34–1.43), and living with friends (aOR 1.39, 95%CI 1.02–1.91) were independent correlates of women’s shame about sexual health-related disorders while living with a spouse (aOR 0.66, 95%CI 0.51–0.86) or children (aOR 0.77, 95%CI 0.62–0.96) were correlated with less shame. Age (aOR 0.98, 95%CI 0.96–0.99) and a postgraduate degree (aOR 0.45, 95%CI 0.28–0.71) were linked with less sexual distress of low sexual desire while having children (aOR 1.38–2.10), intense work pressure (aOR 1.32, 95%CI 1.10–1.60) and heavy family burden (aOR 1.43, 95%CI 1.07–1.92) increased women’s odds of having distress. Women with a postgraduate degree (aOR 0.42, 95%CI 0.19–0.90), more knowledge about sexual health (aOR 0.53–0.67), and decreased sexual desire caused by pregnancy, recent childbirth, or menopausal symptoms (aOR 0.60, 95%CI 0.41–0.85) were less likely to have HSDD, while they were more likely to have HSDD when their decreased sexual desire was due to other sexual issues (aOR 2.56, 95%CI 1.84–3.57) and partners’ sexual problems (aOR 1.72, 95%CI 1.23–2.39).

**Conclusion:**

Sexual health education and related services need to focus on psychological barriers of women with older age, insufficient knowledge of sexual health, intense work pressure, and poor economic conditions. The medical staff need to pay attention to the sexual health of women with intense work or life pressure and a history of gynecological disease. Low sexual desire is not equal to the sexual desire problem, which should be noticed in the future.

**Supplementary Information:**

The online version contains supplementary material available at 10.1186/s12905-023-02357-5.

## Background

Forty years have passed since the rise of Chinese sexual science, especially andrology, in the 1980s [1], but female sexual dysfunction has not been fully diagnosed and treated in China until now. At the end of 2009, the first female sex clinic in Chinese tertiary hospitals began to provide services [2]. However, only less than twenty women sought help there in the first six months. According to Ma, a famous sexologist in China, the ratio of men to women in the sex medicine outpatient clinic of the hospital he worked in is about 17:1 [3]. Many women never seek help for sexual issues or hesitate to do so [4, 5].

There are many reasons for this phenomenon. On the side of patients, another paper of ours based on the health belief model [6] has found that the psychological barrier is the main barrier to seeking medical care for sexual problems in Chinese urban women of childbearing age [7] (under review). More specifically, the relatively lower willingness to communicate sexual health and the significant shame of sexual health-related disorder impeded Chinese women from seeking for treatment of sexual health problems. Although the sexual life and related attitudes of Chinese women have undergone drastic changes to be more liberal since the 1980s [1], the proportion of Chinese women who can talk about “sex” freely is not very high [4, 8–11], and some women are still ashamed of related illness [12]. This may be related to the lack of high-quality comprehensive sex education in the Chinese public in the past forty years [13]. Sex education in China has not kept up with the increasing rates of sexual activity [14], especially education about a positive attitude towards sex and skills to build a good sexual relationship [8]. Such psychological barriers can lead to various behaviors of avoidance, prevent women from gaining valuable health information [15] and enjoying healthy sexual life [16–18], and even result in harmful health outcomes [12, 19], such as delayed treatment of diseases. This not only puts Chinese people at greater risk of STD and HIV infections but also at greater risk of female sexual dysfunction, such as hypoactive sexual desire disorder (HSDD), the most common female sexual dysfunction [20–22].

On the side of medical staff, many doctors and nurses are not well trained to tackle women’s sexual problems [5]. A survey in Tianjin, China, showed that reproductive health medical personnel lacked knowledge of female sexual dysfunction, 90.48% of whom wanted more training for sexual dysfunction [5]. Additionally, the etiology of HSDD hasn’t been fully studied in Chinese women. Doctors may have difficulty in identifying high-risk individuals as well as formulating a proper treatment plan for patients.

Therefore, to facilitate the diagnosis and treatment of HSDD and related female sexual problems, we did this research to accurately locate the target population who are likely to have psychological barriers to sexual health-seeking behaviors and those at risk of HSDD, so that we can provide more targeted sex education and other related services. More specifically, by knowing who is more likely to have psychological barriers, we can provide more information and assistance to help them build a more positive sexual attitude and be more capable to communicate sexual health. By finding who is at a higher risk of HSDD or related symptoms, doctors will be more able to identify high-risk individuals and patients, and thus can intervene earlier to prevent their sexual problems from developing.

### Correlates of women’s psychological barrier to seeking medical care for sexual problems

According to the health belief model [6], women’s psychological barriers can be affected by many modifying factors, such as the sexual health knowledge level, age, related experiences and so on. Research on willingness to communicate sexual health is not much in evidence. We only found some research on sex communication to infer factors that may affect women’s willingness to communicate sexual health. An internet sexual health survey of Chinese women in 2004 [4] shows that better-educated women were more able to talk about their sexual needs. Scheinfeld’s study shows that shame and stigma were significantly and negatively associated with parental and partner communication. The more shame and stigma emerging adults experienced or perceived around STIs, the less likely they were to talk about STIs and sex with their parents, even when in need of social support of varying nature (e.g., financial, informational, emotional) [23]. On the ground of these results, we postulated that Chinese women’s willingness to communicate sexual health with someone close is affected by their level of education, sexual health knowledge, and shame of sexual health-related disorders (Hypothesis 1).

As for risk factors for shame about sexual health-related disorders, there is also limited evidence. A model about the mechanism of the formation of shame posits that shame is related to interpersonal experience in one’s childhood and attachment style [24]. Therefore, Chinese women’s shame about sexual health-related disorders may be associated with the quality of their close relationships. However, because we want to know if there is any overt risk factor that is easy to be noticed, such as demographic features, we choose to conduct an exploratory analysis without making a specific hypothesis.

### Correlates of sexual distress and HSDD

HSDD is characterized by a distressing lack or loss of sexual desire. Research about its related factors in Chinese women is not much in evidence. A survey conducted in Hong Kong, China, shows that women with distressing sexual problems were characterized by having life stressors and having a self-perception that their bodies were unattractive to their boyfriends [21]. As low self-appraisal is usually associated with shame, we postulated that Chinese women’s sexual distress is associated with their shame of sexual health-related disorders (Hypothesis 2).

Women’s sexual distress is usually affected by psychological and social factors, while women’s sexual desire is affected by biological, psychological, and social factors. Therefore, low sexual desire is a heterogeneous symptom, associated with women’s physiological status, the known variability of women’s experience, and the flexibility of their sexuality [25]. As a result, different reasons causing low sexual desire may have different risks for sexual distress and HSDD. However, in many Chinses studies, sexual distress is neglected. Consequently, a low level of sexual desire is conflated with the desire problem, as if women’s sexual desire shouldn’t be low in any context. The fact is that only with sexual distress, can low sexual desire be seen as a symptom of female sexual dysfunction. Otherwise, it may be an adaption to the unsatisfying relationship [26], because many women regard love as the prerequisite of sex. This conflation may cause an overestimation of the prevalence of hypoactive sexual desire disorder in the research and overdiagnosis as well as inappropriate treatment in clinical settings, which has been pointed out in the previous study [27]. Given the heterogeneity of women’s low sexual desire, we postulated that different contributing factors to decreased sexual desire have different risks of having HSDD (Hypothesis 3). By keeping low sexual desire and the desire problem distinct, we can better identify women who are really at risk of HSDD.

Overall, as the sexual health of women of childbearing age has been given growing emphasis in China, the validation of these hypotheses will be helpful to identify women in need of enhanced sexual health attention and those at high risk of HSDD.

## Methods

We conducted an online survey from April to July 2020. Our study was approved by Shanghai Mental Health Center-Institutional Review Board (Number: 2020-05) and was conducted ethically following the World Medical Association Declaration of Helsinki. Public questionnaire links were posted via WeChat, Weibo, and online forums. Voluntary participants were required to read and confirm the informed consent before answering the questions. Demographic characteristics, medical history, sexual health attitudes, and symptoms of HSDD were collected.

### Questionnaire

Demographic characteristics we collected included age, years of education, Minzu, marital status, level of education, occupation, trait, smoking, drinking, family monthly per capita income, monthly household expenditure, work pressure, dominant hand, growth environment, family structure, family relationship, family burden, living with a spouse, living with parents, living with children, living with other relatives, living with friends, living alone, living with other people, number of children, major incidents last year, duration of the stable relationship, physical diseases, and gynecological diseases.

Questions about sexual health attitudes included:

Q1: How knowledgeable are you about sexual health?

Q2: Do you think sexual health-related testing is necessary?

Q3: Do you think sexual health problems are prevalent?

Q4: Do you think treatment is needed for sexual health-related disorders?

Q5: Do you feel sexual health affects one’s partner/couple relationship?

Q6: Are you willing to communicate sexual health with someone close?

Q7: To what extent would you feel ashamed to be diagnosed with a sexual health-related disorder?

Q8: If you were diagnosed with a sexual health-related disorder and would receive treatment at the suggestion of a doctor, you would like to choose: (medication, psychotherapy, or comprehensive treatment).

Except for Q8, the other seven questions were rated on a 5-point Likert scale.

We used the Decreased Sexual Desire Screener (DSDS) [28] to prepare questions about the symptoms of HSDD. The DSDS was designed to diagnose generalized acquired HSDD. Nevertheless, we rewrote the first item as “Are you satisfied with your level of sexual desire or interest recently?“ The complete version of our questions about symptoms of HSDD was as follows:

1. Are you satisfied with your level of sexual desire or interest recently?
2. Has there been a decrease in your level of sexual desire or interest?
3. Are you bothered by your decreased level of sexual desire or interest?
4. Would you like your level of sexual desire or interest to increase?
5. Please circle all the factors that you feel may be contributing to your current decrease in sexual desire or interest:

A. An operation, depression, injuries, or other medical condition.
B. Medications, drugs, or alcohol you are currently taking.
C. Pregnancy, recent childbirth, menopausal symptoms.
D. Other sexual issues you may be having (pain, decreased arousal, or orgasm).
E. Your partner’s sexual problems.
F. Dissatisfaction with your relationship or partner.
G. Stress or fatigue.

A woman would be classified as an HSDD patient if she answered the first item with “No”, the third item with “Yes”, and the fourth item with “Yes”. Of note, our definition differed from the initial version of DSDS. Our rewriting removed the limitation of only identifying patients with the “acquired”. In other words, HSDD of lifelong type would also be identified.

### Statistical analysis

R version 4.1.3 (2022-03-10) was used to describe and analyze data. In total 2575 out of 3443 records (74.8%) were complete. Missing data existed in all variables except the question about the people they live with. Missing data were more common (3.3%~7.1%) in age, years of education, physical diseases, gynecological diseases, and factors contributing to women’s current decrease in sexual desire or interest. Except for these items, missing data were less than 0.5% for most questions, the only exception is the question about the number of children (1.5%). The methods we used to impute numeric data, factor data with 2 levels, and factor data with > 2 levels were predictive mean matching, logistic regression imputation, and polytomous regression imputation, respectively. Although the *mice* package (version 3.14.0) is usually used for multiple imputation, we imputed only once.

A total of 4168 informed consent forms were obtained, with an average response time of 208 s. We excluded (i) response time less than 30 s (n = 13, 0.3%), (ii) age below 18 (n = 61, 1.5%), (iii) responses with no answers to questions about sexual health attitudes and symptoms of HSDD (n = 13, 0.3%), and (iv) male (n = 568, 13.6%) or unknown gender (n = 70, 1.7%). This process left 3443 (82.6%) valid responses.

Binary logistic regression analyses were carried out to examine correlates of Chinese women’s willingness to communicate sexual health with someone close, the shame of sexual health-related disorders, distress due to decreased sexual desire, and HSDD. Those choosing “unwilling” and “not very willing” were categorized as “not willing”, while the rest as “willing”. Those choosing “not ashamed at all” and “not very ashamed” were categorized as “not ashamed”, while the rest as “ashamed”. We used stepwise backward regression by the Akaike information criterion (AIC) and reported variables in the final model. Adjusted odds ratio (aOR) was calculated. P < 0.05 was regarded as statistically significant.

## Results

### Characteristics of the sample

Table 1 shows the characteristics of our sample, who were mainly Chinese urban women of childbearing age (median 26 years old, Q1-Q3 23–30). They were distributed in all of China’s provincial administrative regions, except for Tibet and Taiwan.

**Table 1:** Demographics of participants

		(Continued)		(Continued)	
Variables	N(%)/M(Q1-Q3)	Variables	N(%)/M(Q1-Q3)	Variables	N(%)/M(Q1-Q3)
		**Minzu**		**Occupation**	
**Age**	26 (23–30)	Han	3268 (94.9%)	Farmers	84 (2.4%)
Missing	204 (5.9%)	Others	35 (5.0%)	Workers	206 (6.0%)
**Marital status**		Missing	4 (0.1%)	Employees	1243 (36.1%)
Unmarried	1482 (43.0%)	**Years of education**		Civil servants	391 (11.4%)
Married	1776 (51.6%)	median (Q1-Q3)	15 (12–16)	Professional	627 (18.2%)
Widowed	66 (1.9%)	Missing	120 (3.5%)	Private individuals	182 (5.3%)
Divorced	82 (2.4%)	**Education**		Retired	39 (1.1%)
Separated	32 (0.9%)	Illiterate	27 (0.8%)	Students	589 (17.1%)
Missing	5 (0.1%)	Elementary school	50 (1.5%)	Unemployed	37 (1.1%)
**Duration of stable relationship**		Junior high school	230 (6.7%)	Others	43 (1.2%)
≥ 12 months	2078 (60.4%)	High school	551 (16.0%)	Missing	2 (0.1%)
6–12 months	688 (20.0%)	University	2122 (61.6%)	**Monthly household income per capita**	
1–6 months	627 (18.2%)	Postgraduate	459 (13.3%)	≥¥5000	1881 (54.6%)
Missing	50 (1.5%)	Missing	4 (0.1%)	¥3000-¥5000	1158 (33.6%)
**Number of children**		**Growth environment**		¥1000-¥3000	330 (9.6%)
0	1673 (48.6%)	Tier 1 cities	743 (21.6%)	<¥1000	66 (1.9%)
1	1122 (32.6%)	Tier 2 cities	1228 (35.7%)	Missing	8 (0.2%)
2	523 (15.2%)	Tier 3 and below	993 (28.8%)		
> 2	119 (3.5%)	Rural areas	475 (13.8%)		
Missing	6 (0.2%)	Missing	4 (0.1%)		

**Fig. 1:**
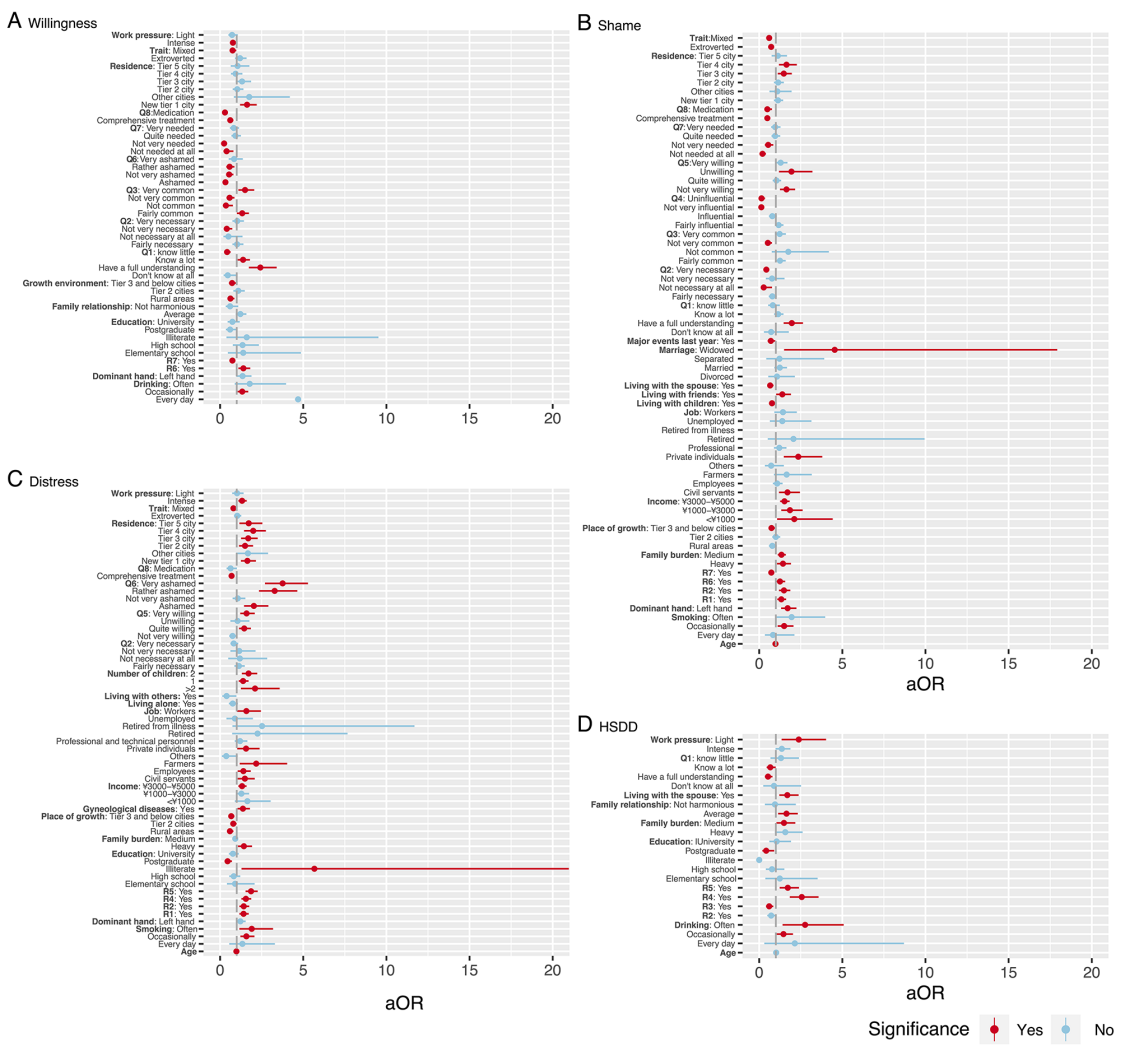
Correlates of variables of interest A-D showed the statistically significant variables in the four final models. Q1-Q8 referred to the questions of sexual attitudes questionnaire. R1-R7 referred to the reasons for women’s decrease in sexual desire in our questionnaire about symptoms of HSDD. The upper limit of the 95% confidence interval of “Illiterate” in Fig. 1C was 42.07

### Correlates of women’s psychological barrier to seeking medical care for sexual problems

Figure 1 A shows all the independent variables in the final model with the willingness to communicate sexual health as the dependent variable. Knowing a lot (aOR 1.38, 95%CI 1.06–1.79, compared with “know”) or having a full understanding of sexual health knowledge (aOR 2.42, 95%CI 1.73–3.40) was correlated with a higher willingness to communicate sexual health while knowing little about sexual health knowledge (aOR 0.42, 95%CI 0.28–0.63, compared with “know”) and shame of sexual health-related disorders (aOR 0.32–0.57, compared with “not ashamed at all”) were correlated with lower willingness.

As for Chinese women’s shame of sexual health-related disorders (Fig. 1B), correlates of more shame included monthly household per capita income below ¥5000 (aOR 1.52–2.11, compared with “>¥5000”), living in a tier 3 city or a tier 4 city (aOR 1.48–1.64, compared with “tier 1 city”), having medium (aOR 1.34, 95%CI 1.12–1.60) or heavy family burden (aOR 1.43, 95%CI 1.07–1.91, compared with “light”), living with friends (aOR 1.39, 95%CI 1.02–1.91, compared with “not living with friends”), and having a full understanding of sexual health knowledge (aOR 1.96, 95%CI 1.47–2.63, compared with “know”). Correlates of less shame included living with a spouse (aOR 0.66, 95%CI 0.51–0.86, compared with “not “) or children (aOR 0.77, 95%CI 0.62–0.96, compared with “not”).

**Fig. 2:**
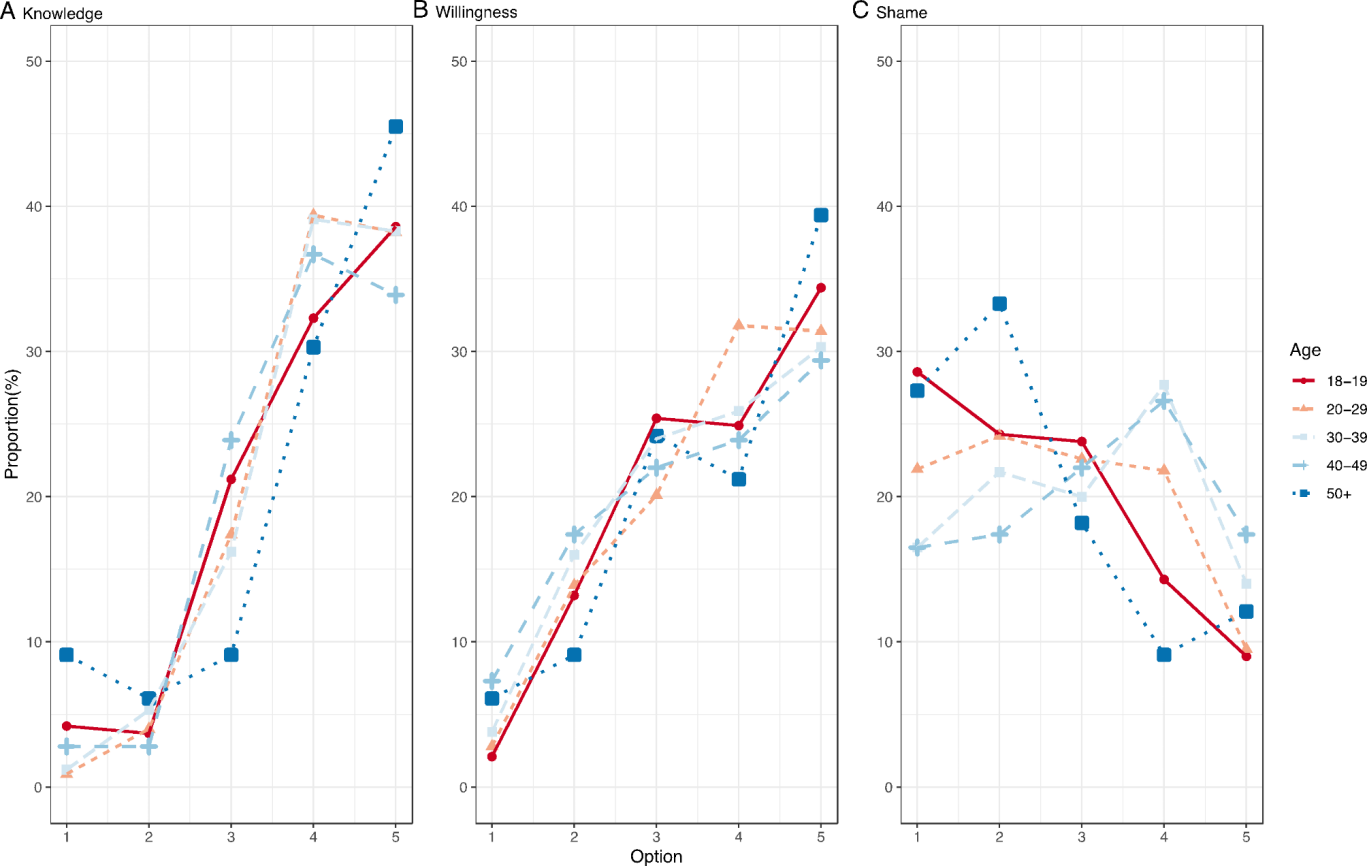
Differences in key variables by age group A: The perceived of sexual health knowledge level; B: Willingness to communicate sexual health; C: Shame of sexual health-related disorders, N = 3443. Option 1 to option 5 refers to the 5-point Likert options of our questions about women ’s perceived sexual health knowledge level and their sexual health attitudes. From option 1 to option 5, the degree of positivity of the responses decreases. For example, women who chose option 1 were least willing to communicate sexual health and most ashamed of sexual health-related disorders.

The stepwise regression analysis excluded *education* in the final model of women’s shame, while the final model of willingness included it but showed no statistically significance and excluded *age*. In terms of the common sense, education and age have a great impact on people’s attitudes. To understand the phenomenon behind the results, we drew another two figures. Figure 2 shows the results of stratification by age on the perceived of sexual health knowledge level (Q1, Fig. 2A), the willingness to communicate sexual health with close people (Q5, Fig. 2B), and the shame of sexual health-related disorders (Q6, Fig. 2C). The perceived sexual health knowledge level and the willingness to communicate with close people about sexual health in different age groups were generally similar, but there were obvious differences in the shame of sexual health-related disorders. Among the respondents in this study, women over 50 years old and women aged 18–19 years were most ashamed of sexual health-related disorders, while women aged 30–49 years were least ashamed of sexual health-related disorders.

**Fig. 3:**
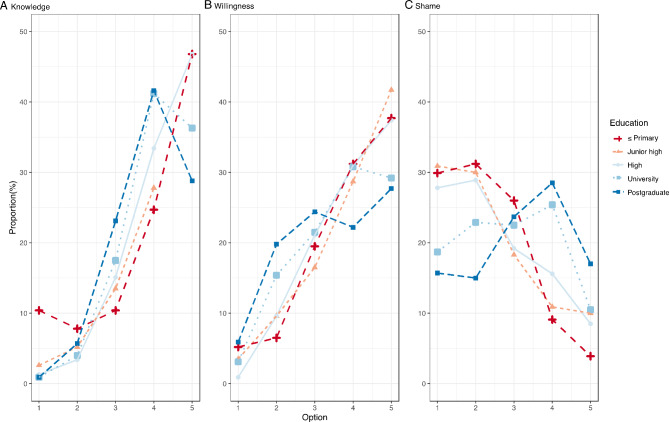
Differences in key variables for different education level groups A: The perceived of sexual health knowledge level; B: Willingness to communicate sexual health; C: Shame of sexual health-related disorders, N = 3443. Option 1 to option 5 refers to the 5-point Likert options of our questions about women ’s perceived sexual health knowledge level and their sexual health attitudes. From option 1 to option 5, the degree of positivity of the responses decreases. For example, women who chose option 1 were least willing to communicate sexual health and most ashamed of sexual health-related disorders.

Figure 3 shows the results of stratification by education level on the perceived sexual health knowledge level (Q1, Fig. 3A), the willingness to communicate sexual health with close people (Q5, Fig. 3B), and the shame of sexual health-related disorders (Q6, Fig. 3C). Compared with other groups, the self-reported sexual health knowledge level of women who complete college or postgraduate education was more conservative (more concentrated towards the middle level). These women’s willingness to communicate on sexual health was lower than that of women with other levels of education, as well as their shame about health-related disorders. From Fig. 3C, we can see that women who only finished junior high school education or have not completed junior high school education were most ashamed of sexual health-related disorders.

### Correlates of sexual distress and HSDD

In terms of Chinese women’s distress associated with decreased sexual desire (Fig. 1C), a postgraduate degree (aOR 0.45, 95%CI 0.28–0.71, compared with “junior high school”) was correlated with less distress. The shame of sexual health-related disorders (OR 2.03–3.76, compared with “not ashamed at all”), monthly household per capita income between ¥3000 and ¥5000 (aOR 1.32, 95%CI 1.10–1.60, compared with “more than ¥5000”), living in cities other than tier 1 cities (aOR 1.50–1.99, compared with “tier 1 city”), heavy family burden (aOR 1.43, 95%CI 1.07–1.92, compared with “light”) correlated with more distress. The lack of sexual desire was more likely to bother women whose decreased desire was due to an operation, depression, or another medical condition (aOR 1.41, 95%CI 1.16–1.72, compared with “no”), or substances (medications, drugs, or alcohol, aOR 1.42, 95%CI 1.16–1.75, compared with “no”). A woman was more likely to feel distressed when her decreased sexual desire was due to other sexual issues (aOR 1.55, 95%CI 1.28–1.87, compared with “no”) or her partner’s sexual problems (aOR 1.86, 95%CI 1.53–2.26, compared with “no”).

When it comes to HSDD (Fig. 1D), those with decreased sexual desire due to pregnancy, recent childbirth, or menopausal symptoms were less likely to be HSDD patients (aOR 0.60, 95%CI 0.41–0.85). However, a woman with decreased sexual desire due to other sexual issues (aOR 2.56, 95%CI 1.84–3.57) and her partner’s sexual problems (aOR 1.72, 95%CI 1.23–2.39) was more likely to be HSDD patients. Occasional (aOR 1.46, 95%CI 1.04–2.03, compared with “never”) or frequent (aOR 2.76, 95%CI 1.41–5.08) drinking, and living with a spouse (aOR 1.69, 95%CI 1.21–2.37, compared with “no”) also increased women’s odds of having HSDD.

## Discussion

Our results identified characteristics of women in need of enhanced sexual health attention and those at risk of HSDD and supported most of the hypotheses we proposed. Women’s perceived sexual health knowledge level was correlated with a higher willingness to communicate sexual health, while shame about sexual health-related disorders was correlated with a lower willingness (partially supported hypothesis 1). Correlates of more sexual distress included shame about sexual health-related disorders (supported hypothesis 2). Women with decreased sexual desire due to childbirth or menopausal symptoms were less likely to have HSDD, which was distinct from decreased sexual desire due to women’s other sexual problems and partner sexual problems (supported hypothesis 3). More details are discussed in the next paragraphs.

### Correlates of Chinese urban women’s willingness to communicate sexual health

The psychological barrier is the main barrier to Chinese urban women’s sexual health-related help-seeking behaviors. Therefore, exploring the characteristics of women who are at high risk of psychological barriers is of great significance to provide targeted services. Our results showed women with a more positive perception of their sexual health knowledge level were more willing to communicate sexual health. However, a postgraduate degree (OR 0.60, 95% CI 0.35–1.01, p = 0.061, compared with “junior high school”) was potentially related to lower willingness, which was inconsistent with our hypothesis 1. Our hypothesis 1 was based on an internet survey conducted by Ma in 2004 [4], the sample of which was similar to ours. "The difference between our results may be caused by the following reasons: (1) Ma’s report only statistically described the phenomenon without controlling confounding factors or conducting further analysis; (2) the main outcome indicator of Ma’s study was sexual communication behavior rather than the willingness to communicate sexual health. Our results were different from the general impression that women with more education are more willing to communicate sexual health. This may reflect the insufficient attention to sex education, especially about sexual relations [8] in the general education system [13]. According to *International technical guidance on sexuality education* published by UNESCO in 2018, comprehensive sexuality education not only includes sexual health knowledge, but emphasizes helping students develop sexuality knowledge, skills, and attitudes, so as to enjoy positive sexuality and good sex. Therefore, future research can further focus on the implementation of attitude and skill education in sex education in China and its impact on sexual health promotion behavior.

In terms of other elements of the sexual health belief model [7], the related factors of sexual health communication willingness included the belief that sexual health-related testing was not very necessary (linked with lower willingness), the awareness of the prevalence of sexual health problems (the perception of uncommonness was linked with lower willingness, the perception of commonness was linked with higher willingness), the shame about sexual health-related disorders (linked with lower willingness), and the perception that sexual health-related disorders didn’t require treatment (linked with lower willingness). Based on this model, our results indicated that people who recognized their susceptibility to sexual problems had a lower risk of psychological barriers, while those who had insufficient awareness of the benefits of treatment were at higher risk of psychological barriers. Additionally, the awareness of severity was irrelevant to women’s willingness to communicate sexual health. The relationship between the perception of susceptibility to sexual problems and psychological barriers may reflect the special “interpersonal characteristic” of sexual health. For many diseases, the main driving force for people to seek medical services and obey treatment is the severity of these diseases and the benefit of treatment, and barriers are always related to the convenience and costs [29, 30]. However, when it comes to sexuality, concerns about how they will be perceived by others have a greater impact on communication [31]. Because of this, awareness of the prevalence of sexuality helps alleviate women’s interpersonal stress and anxiety by making the problem universal. This was also reflected in the correlation between shame and willingness to communicate. In terms of the relationship between women’s willingness to communicate sexual health and their perception of treatment benefits, one possible explanation is that people with higher psychological barriers may rationalize their avoidance tendencies by ignoring the benefits of treatment for sexual health-related disorders. Taken together, these results highlight the need to pay more attention to sexual health attitudes and skills in sex education and related services.

High work pressure and low sexual desire or interest due to stress or fatigue were linked with lower willingness to communicate on sexual health among urban women of childbearing age in China. This result is consistent with common sense, suggesting that sexual health services should not only be limited to sex but also expand their focus on women’s overall living conditions and physical and mental health. Another interesting phenomenon was that Chinese urban women who live in a new tier 1 city (compared to a tier 1 city) were more willing to communicate sexual health. To explore the reasons behind this phenomenon, we included *monthly household income per capita* and *family burden* into the model to control the confounding factors and found that residence in a new tier 1 city (OR 1.58, 95% CI 1.17–2.15, p = 0.003, compared with “tier 1 city”) was still linked with higher willingness to communicate sexual health. Does this mean that Chinese urban women of childbearing age who choose to live in new tier 1 cities place more emphasis on sexual health and partnerships, while women living in tier 1 cities place more emphasis on production and consumption? Growing up in tier 3 cities and below cities or in rural areas was linked with a lower willingness to communicate on sexual health, which may be related to the relatively conservative sexual attitudes in those women’s growth environment. On this basis, future research can further validate the relationship between these two demographic variables and sexual health attitudes and explore the underlying reasons for their relationship.

### Correlates of Chinese women’s shame about sexual health-related disorders

Shame is the biggest psychological barrier impeding Chinese urban women’s sexual health-seeking behavior. Therefore, women feeling ashamed of sexual health-related disorders should be the focus of sexual health services. Our results showed that the correlates of more shame about sexual health-related disorders included age, widow, occasional or frequent smoking, left-hander, occupation of civil servants or private individuals (compared with students), current residence in tier 3 or tier 4 cities (compared with tier 1 cities), monthly household income per capita less than ¥5,000, and moderate or heavy family burden. Most of these variables were related to economic status, indicating that socioeconomic status was closely related to women’s shame about sexual health-related disorders. As for the underlying reason for the correlation, it may not only be related to differences in sexual concepts of women with different economic statuses. There is a possibility that socioeconomic status is related to women’s shame by affecting how women view themselves. These results suggested that sexual health-related services need to pay more attention to low-income women and be alert to possible cross-stigma related to economic status. Interestingly, living with friends was linked with more shame of sexual health-related disorders but living with a spouse or children wasn’t. There are different possible reasons: (1) women may have a stronger sense of being gazed at or judged when living with friends, thus have more shame of sexual health-related disorders [32]; (2) those living with friends may have sex outside of “acceptable” social boundaries and thus experience shame, unlike women living with a husband having sex in an accepted manner. As we didn’t ask women about their sexual behavior, to test the assumptions mentioned above, the relationship between living with friends and shame needs further exploration.

In terms of other elements in the sexual health belief model [7], more perceptive sexual health knowledge and the reluctance to communicate sexual health were linked with more shame. Less shame was seen if one underestimated the necessity of sexual health-related testing and treatment, the prevalence of sexual health problems, and the impact of sexual health on partnerships. This finding suggested that women who underestimated the susceptibility and severity of sexual health-related disorders, as well as the benefit of treatment, might be less likely to experience related shame. Namely, awareness of susceptibility to sexual problems was inversely associated with shame and willingness to communicate. Women who perceived sexual health problems as uncommon were at higher risk of unwillingness to communicate sexual health, but lower risk of shame. This phenomenon may indicate that shame and communication pressure were psychological barriers of different causes. For example, shame about a diagnosis may be related to people’s perceptions of “disease”, while willingness to communicate sexual health may not. Therefore, the lower risk of shame for women with a low evaluation of severity and treatment benefit may be because having this disease will not be a big deal to them. Since it is not serious, there is no shame.

A special phenomenon was that recognizing sexual health-related testing as unnecessary and as very necessary were both linked with less shame about sexual health-related disorders. The correlation of feeling “not necessary at all " with less shame can be explained by the assumptions mentioned above, while the effect of feeling “very necessary” may be because women with less shame are more willing to screen sex issues. Another possible explanation is that participants’ different understandings of “sexual health-related testing” lead to seemingly contradictory results because we haven’t fully validated our questionnaire in a pilot study. Based on the above discussion, although some women are not very ashamed, their neglect of the susceptibility, severity, and treatment benefit of sexual problems can also cause adverse health outcomes. Therefore, increasing women’s understanding of sexual health-related disorders is also an important part of sexual health services. Perception of having a full understanding of sexual health knowledge was linked with more shame. This contradicted our understanding that more knowledge led to higher awareness of sexual rights and the barriers to sexual health. The separation of perceived sexual health knowledge level and a more liberal attitude might result from the ignorance of attitude education in school sexuality education [10, 13, 33, 34] and the low quality of internet content on this issue [13, 15, 33, 34].

### Correlates of Chinese women’s sexual distress of decreased sexual desire

The exploration of correlates of sexual distress can not only help to identify high-risk individuals with HSDD but also promote research on the etiology of this disease. A lower degree of shame was linked with less sexual distress, which supported our hypothesis 3 and the speculation of the previous study [35]. Those more willing to communicate sexual health were more likely to have sexual distress due to low sexual desire. This result seemed strange at a glance because some research suggested sexual communication can help to alleviate distress and promote problem-solving [36, 37]. There are several possibilities for this phenomenon. First, Chinese women may attempt to gain stronger control of their sexual lives and are holding higher sexual expectations [4, 20], so they are more willing to communicate, and their distress is caused by the mismatch between reality and their expectations. The second possibility is that if women don’t have much experience in promoting constructive communication and are used to criticizing and complaining, although they are willing to communicate sexual health with their husbands, the communication can be disappointing for the couple and only serves to exacerbate women’s distress. Third, if women are willing to communicate sexual health with friends instead of their partners, peer pressure may cause them to feel distressed by more guilt and self-criticism. These possibilities also emphasize the importance of education about positive attitudes towards sex and skills to build a good sexual relationship.

Additionally, those with a decrease in sexual desire due to an operation, depression or other medical conditions, substances (medications, drugs, or alcohol), other sexual issues, or their partner’s sexual problems were more likely to be distressed. These all reflected the complexity of sexual distress and suggested that doctors should pay more attention to decreased sexual desire caused by medical conditions and consider the sexual health of women’s partners when formulating the treatment plan. History of gynecological disease was also correlated with more sexual distress. This suggested that gynecologists especially need to increase their attention to the sexual issues of female patients and refer patients to psychiatrists or clinical psychologists in time for further treatment if necessary.

The correlations between sexual distress and intense work pressure, heavy family burden, and a low education level were consistent with the previous study [21, 38]. This demonstrated the imperative to pay more attention to these groups. Considering the relationship between sexual distress and shame, whether the distress of these women is caused by sexual objections and low self-appraisal [39] deserves further exploration.

### Correlates of HSDD in Chinese women

The exploration of correlates for HSDD can help us to identify the women who need help among those with decreased sexual desire. Whose decreased sexual desire was due to pregnancy, recent childbirth, and menopausal symptoms were relatively less likely to be HSDD patients than those due to other sexual issues or partners’ sexual problems. This difference demonstrated the heterogeneity of women’s low sexual desire (Hypothesis 3). Low sexual desire is not equal to the sexual desire problem. For example, a couple may reach an agreement on avoiding sexual behaviors due to the consideration of staying safe while pregnant. The labor and housework after childbirth may also reduce the priority of sexual conflict between partners. For this reason, it’s necessary to clarify the reasons for the decrease in sexual desire and consider sexual distress when investigating the sexual desire problem in research and clinical settings, instead of regarding low sexual desire as sexual dysfunction simplistically.

Age was not related to the risk of HSDD, which was consistent with the previous study [40]. High education and more understanding of sexual health were linked with a lower risk of HSDD. This demonstrated education’s role in promoting female sexual health and was consistent with the relationship between education and sexual distress. In addition, drinking was linked with a higher risk of HSDD, but not with the risk of distress. It meant that drinking might be related to low sexual desire. As we didn’t evaluate sexual desire online, the relationship between alcohol and HSDD in Chinese women deserved further exploration in the future. Interestingly, although intense work pressure and heavy family burden were related to a higher risk of sexual distress, the factors related to a higher risk of HSDD were an average family relationship, light work pressure, and medium family burden. One possibility is that only when the work pressure and family burden of women are not too heavy and the family relationship not too bad, do they have the wish and motivation to improve their sexual life. For those with a heavy family burden and intense work pressure, although they may have distress, their symptoms may not reach a diagnostic level because their wish and motivation to improve their sexual life have been diminished by the more overwhelming realistic problems. Before they can make efforts to be happy, they need to strive to survive.

### Limitations

Our conclusions did come with many caveats. First, response bias was an inherent part of an online survey. Our convenient sample may not represent Chinese urban women well in many aspects, such as age, marital status, and internet accessibility. To reduce bias and increase representativeness, we distributed our questionnaire as more as possible and attained a relatively broad geographical distribution. Second, our outcomes were self-reported and not validated. Respondents may have different interpretations of the questions. To deal with this issue, we introduced some facts about female sexual dysfunction in our informed consent to avoid misunderstanding as much as possible. Third, our definition of HSDD was somewhat different from DSM-IV and the original version of DSDS. When diagnosing, eliminating the fifth question (factors to be ruled out) of DSDS and considering only the first four questions increased its sensitivity slightly but impaired specificity. This may prevent us from discovering all correlates of HSDD. However, our data can support our hypotheses. Despite all these limitations, we still provided valuable information and contributed to the profile of Chinese women’s sexuality.

## Conclusions

To facilitate the diagnosis and treatment of hypoactive sexual desire disorder and related female sexual problems, sexual health education and related services need to pay attention to the psychological barriers of women with older age, insufficient knowledge of sexual health, intense work pressure, and poor economic conditions. Medical staff in the sexual health-related department need to pay attention to the sexual health of women with the intense pressure of work and life and history of gynecological disease.

Moreover, low sexual desire only due to pregnancy, childbirth, or menopausal symptoms was linked with a lower risk of HSDD, while other common causes were not. Therefore, low sexual desire is not equal to the sexual desire problem. The heterogeneity of low sexual desire should be noticed in future research. These findings also highlighted the need to further explore the complexity of women’s sexual desire, to improve couples’ sexual health.

## Electronic supplementary material

Below is the link to the electronic supplementary material.

Supplementary Material 1: Variables in the final logistic regression models

## Data Availability

The datasets generated and/or analyzed during the current study are not publicly available due to the protection of the privacy of our participants but are available from the corresponding author on reasonable request.
