# Supplementary material for: Characteristics of Chinese women in need of enhanced sexual health attention and at risk of hypoactive sexual desire disorder
Source: BMC Womens Health. 2023 Jun 13;23:307. doi: 10.1186/s12905-023-02357-5 (PMC10265816; doi:10.1186/s12905-023-02357-5)
Supplement: Supplementary file 1 — Supplementary Material 1: Variables in the final logistic regression models [file 12905_2023_2357_MOESM1_ESM.docx]

Supplemental Table 1. Demographic variables in the final regression models

| Variable | N |  |  | **Family relationship** | | |
| --- | --- | --- | --- | --- | --- | --- |
| **Trait** | | |  | Harmonious | 2670 | Ref |
| Introverted | 890 | Ref |  | Average | 682 |  |
| Extroverted | 1232 |  |  | Not harmonious | 91 |  |
| Mixed | 1321 |  |  | **Family burden** | | |
| **Smoking** | | |  | Light | 1247 | Ref |
| Never | 2854 | Ref |  | Medium | 1797 |  |
| Occasionally | 425 |  |  | Heavy | 399 |  |
| Often | 132 |  |  | **Living with spouse** | | |
| Every day | 32 |  |  | No | 1774 | Ref |
| **Drinking** | | |  | Yes | 1669 |  |
| Never | 2377 | Ref |  | **Living with children** | | |
| Occasionally | 915 |  |  | No | 2142 | Ref |
| Often | 131 |  |  | Yes | 384 |  |
| Every day | 20 |  |  | **Living with friends** | | |
| **Work pressure** | | |  | No | 3098 | Ref |
| Moderate | 2063 | Ref |  | Yes | 345 |  |
| Intense | 1123 |  |  | **Living alone** | | |
| Light | 257 |  |  | No | 3188 | Ref |
| **Dominant hand** | | |  | Yes | 255 |  |
| Right hand | 2863 | Ref |  | **Living with others** | | |
| Left hand | 580 |  |  | No | 3399 | Ref |
| **Residence** | | |  | Yes | 44 |  |
| Tier 1 cities | 745 | Ref |  | **Major events last year** | | |
| New Tier 1 cities | 788 |  |  | No | 3215 | Ref |
| Tier 2 cities | 723 |  |  | Yes | 228 |  |
| Tier 3 cities | 543 |  |  | **Gynecological diseases** | | |
| Tier 4 cities | 373 |  |  | No | 3008 | Ref |
| Tier 5 cities | 191 |  |  | Yes | 435 |  |
| Others | 80 |  |  |  |  |  |

Supplemental Table 2. Non-demographic variables in the final regression models

| Variable | N |  |  | Ashamed | 756 |  |
| --- | --- | --- | --- | --- | --- | --- |
| ***Sexual health attitudes*** | | |  | Rather ashamed | 807 |  |
| **Q1: Perception of knowledge about sexual health** | | |  | Very ashamed | 716 |  |
| Know | 600 | Ref |  | **Q7: Perceptions of the degree to which the disease requires treatment** | | |
| Don’t know at all | 45 |  |  | Need | 608 | Ref |
| know little | 148 |  |  | Not needed at all | 71 |  |
| Know a lot | 1335 |  |  | Not very needed | 132 |  |
| Have a full understanding | 1315 |  |  | Quite needed | 1201 |  |
| **Q2: Perceptions of the necessity of sexual health-related testing** | | |  | Very needed | 1431 |  |
| Necessary | 485 | Ref |  | **Q8: Treatment preferences for sexual health-related disorders** | | |
| Not necessary at all | 38 |  |  | Psychotherapy | 770 | Ref |
| Not very necessary | 70 |  |  | Medication | 136 |  |
| Fairly necessary | 1097 |  |  | Comprehensive treatment | 2537 |  |
| Very necessary | 1753 |  |  | ***Reasons for women's decrease in sexual desire*** | | |
| **Q3: Perceptions of the prevalence of sexual health problems** | | |  | **R1: An operation, depression, injuries, or other medical condition** | | |
| Common | 728 | Ref |  | No | 2297 | Ref |
| Not common | 50 |  |  | Yes | 1146 |  |
| Not very common | 158 |  |  | **R2: Medications, drugs, or alcohol you are currently taking** | | |
| Fairly common | 1209 |  |  | No | 2536 | Ref |
| Very common | 1298 |  |  | Yes | 907 |  |
| **Q4: Perceptions of the degree to which sexual health influences partner relationship** | | |  | **R3: Pregnancy, recent childbirth, menopausal symptoms** | | |
| Influential | 1530 | Ref |  | No | 2410 | Ref |
| Uninfluential | 45 |  |  | Yes | 1033 |  |
| Not very influential | 88 |  |  | **R4: Other sexual issues you may be having** | | |
| Fairly influential | 577 |  |  | No | 2071 | Ref |
| Very influential | 1203 |  |  | Yes | 1372 |  |
| **Q5: Willingness to communicate sexual health** | | |  | **R5: Your partner’s sexual problems** | | |
| Willing | 736 | Ref |  | No | 2366 | Ref |
| Unwilling | 110 |  |  | Yes | 1077 |  |
| Not very willing | 497 |  |  | **R6: Dissatisfaction with your relationship or partner** | |  |
| Quite willing | 1021 |  |  | No | 2499 | Ref |
| Very willing | 1079 |  |  | Yes | 944 |  |
| **Q6: Shame of sexual health-related disorders** | | |  | **R7: Stress or fatigue** | |  |
| Not ashamed at all | 373 | Ref |  | No | 1536 | Ref |
| Not very ashamed | 791 |  |  | Yes | 1907 |  |
